# Integrase and integration: biochemical activities of HIV-1 integrase

**DOI:** 10.1186/1742-4690-5-114

**Published:** 2008-12-17

**Authors:** Olivier Delelis, Kevin Carayon, Ali Saïb, Eric Deprez, Jean-François Mouscadet

**Affiliations:** 1 LBPA, CNRS, Ecole Normale Supérieure de Cachan, 61 Avenue du Président Wilson, 94235 Cachan, France; 2 CNRS, Hôpital Saint-Louis, 1 Avenue Claude Vellefaux, 75475 Paris Cedex 10, France

## Abstract

Integration of retroviral DNA is an obligatory step of retrovirus replication because proviral DNA is the template for productive infection. Integrase, a retroviral enzyme, catalyses integration. The process of integration can be divided into two sequential reactions. The first one, named 3'-processing, corresponds to a specific endonucleolytic reaction which prepares the viral DNA extremities to be competent for the subsequent covalent insertion, named strand transfer, into the host cell genome by a trans-esterification reaction. Recently, a novel specific activity of the full length integrase was reported, *in vitro*, by our group for two retroviral integrases (HIV-1 and PFV-1). This activity of internal cleavage occurs at a specific palindromic sequence mimicking the LTR-LTR junction described into the 2-LTR circles which are peculiar viral DNA forms found during viral infection. Moreover, recent studies demonstrated the existence of a weak palindromic consensus found at the integration sites. Taken together, these data underline the propensity of retroviral integrases for binding symmetrical sequences and give perspectives for targeting specific sequences used for gene therapy.

## Background

The human immunodeficiency virus is the causal agent of AIDS. AIDS morbidity and mortality have led to efforts to identify effective inhibitors of the replication of this virus. Viral replication is driven by a molecular motor consisting of the three viral enzymes: the reverse transcriptase, protease and integrase (IN). The genomic RNA of the virus is used to produce a copy of viral DNA by reverse transcription, and the last of these enzymes, integrase, catalyses the covalent insertion of this DNA into the chromosomes of the infected cells. Once integrated, the provirus persists in the host cell and serves as a template for the transcription of viral genes and replication of the viral genome, leading to the production of new viruses. Integrase possesses two major catalytic activities: an endonucleolytic cleavage at each 3'-OH extremities of the viral genome, named 3'-processing, and a strand transfer reaction leading to the insertion of the processed viral DNA into the target DNA by a trans-esterification mechanism. These catalytic functions of the integrase are essential for the overall integration process and have thus been the object of intensive pharmacological research. Since the end of the 1990s, several inhibitors with genuine antiviral activity have been identified and developed. Two of these compounds – MK-0518 or raltegravir and GS9137 or elvitegravir – have shown great promise and should ensure that integrase inhibitors rapidly become an important class in the arsenal of antiretroviral drugs (ARVs) available [1]. In addition to 3'-processing and strand transfer, IN may efficiently catalyse other reactions: a third reaction, named disintegration, corresponds to the apparent inverse reaction of the strand transfer [2] although it is not clear whether it may occur in the cell context. More recently, a specific and internal cleavage catalysed by the full-length IN has been observed *in vitro *[3]. This reaction requires a symmetrical organisation of the DNA substrate as well as a tetrameric organisation of the protein. From a structural point of view, this reaction is related to the endonucleolytic reaction of a restriction enzyme.

*In vivo*, the integrase oligomer and viral DNA molecule form part of a preintegration complex (PIC), our knowledge of which remains limited. The reverse transcriptase (RT), matrix protein (MA), Vpr and the nucleocapsid protein (NC) are also present in this complex as well as cellular partners [4-7]. The presence of an intact integrase is required for the stabilisation of preintegration complexes and their transport into the nucleus: These non catalytic functions of IN are also crucial for the viral replication cycle. Indeed, a functional interaction between IN and RT has been observed, suggesting that IN is involved, at least indirectly, in controlling the synthesis of viral DNA [8-10]. Furthermore, the interaction of particular IN structures with one or several cellular cofactors plays a key role for the integration into host cell chromosomes. For instance, LEDGF/p75 acts as a chromatin tethering factor for IN [11,12]. All these observations pave the way for the development of inhibitors targeting the interactions between IN and either viral or cellular cofactors. These alternative functions may constitute useful targets for the future development of integrase inhibitors.

### Integrase

Integrase is a 288-amino acid protein (32 kDa) encoded by the end of the *pol *gene. It is produced as part of the Gag-Pol polypeptide precursor, from which it is released by viral protease-mediated cleavage. It has three independent domains: (i) The N-terminal domain (amino acids 1–49) that carries an HHCC motif analogous to a zinc finger, and effectively binds Zn^2+ ^[13], possibly favouring protein multimerisation, a key process in integration [13,14]. (ii) The central domain or catalytic domain (amino acids 50–212) encompassing a D, D-35, E motif which is indispensable for the catalytic activity and which is conserved between viral IN and transposases. This central domain is also implicated in the binding of the viral DNA extremities mainly via the residus Q148, K156 and K159 [15-19]. All integrase activities strictly require the presence of a metallic cationic cofactor which is coordinated by two residues of the catalytic triad (D64 and D116 for HIV-1 IN) [20,21]. (iii) The C-terminal domain (amino acids 213–288) binds non-specifically to DNA and therefore is mainly involved in the stability of the complex with DNA. No complete structure has yet been determined for the integrase protomer (IN^1–288^), or for oligomers or complexes of these structures with DNA, due to poor solubility and interdomain flexibility problems. However, several structures of isolated domains or of two consecutive domains have been reported [20-25].

Integrase functions in a multimeric form, as shown by complementation experiments: mixtures of proteins, each individually inactive, were found to be active [26-28]. For example, an inactive catalytic triad mutant can be complemented by an inactive integrase truncated at its C-terminal end. Such a functional complementation can be observed in virions [29]. In addition, the factors promoting integrase multimerisation such as Zn^2+ ^also stimulate the specific Mg^2+^-dependent activity of the enzyme [14], indicating that functional enzyme is multimeric. Dimers form at either end of the viral DNA molecule. These dimers are responsible for 3'-processing activity [30-34]. Pairs of dimers bring together the two ends of the viral DNA and leads to the formation of a tetramer (dimer of dimer), the active form for concerted integration [35,36]. During its catalytic cycle, IN must bind simultaneously to the viral substrate DNA and the target DNA. Current knowledge of the organisation of this tetramer onto DNA is based exclusively on models constructed from partial structural and biochemical (cross-linking and site-directed mutagenesis) data [24,37-40]. In a recent model, an IN tetramer is bound to the two ends of the viral DNA, *i.e. *LTRs (Long Terminal Repeat), and to a 26 base pairs host DNA molecule in the presence of Mg^2+ ^[40]. This model takes into account the structural constraints deduced from the model of the complex formed between DNA and a related enzyme, the Tn5 transposase, and the observation that the two ends of the viral DNA are integrated five base pairs apart, corresponding to a distance of about 16 Å. This model may provide a platform for the rational design of new inhibitors. It is important to note that most of these models support a symmetrical form of IN for concerted integration. However, recently, Ren *et al. *have proposed an asymmetric tetramer/DNA model for the concerted integration suggesting that at least a reaction intermediate could be asymmetric [39].

### The catalytic activities of integrase (IN)

#### 3'-processing and strand transfer

**Figure 1:**
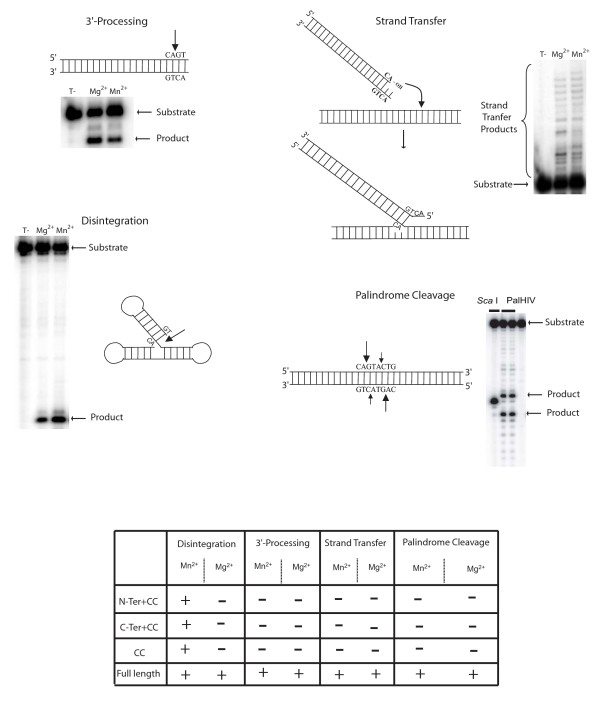
Catalytical activities of HIV-1 integrase. The catalytical activities 3'-processing (A), strand-transfer reaction (B), disintegration (C) and palindrome cleavage (D) are represented. The domains of the protein responsible for these activities are depicted in the table above.

There is now substantial virological evidence that the precursor of integrated viral DNA, or provirus, is a linear viral DNA generated by reverse transcription of the viral genome. Two reactions are required for the covalent integration of viral DNA into the host DNA. The integrase (IN) first binds to a short sequence at each end of the viral DNA known as the long terminal repeat (LTR) and catalyses an endonucleotide cleavage known as 3'-processing, in which a dinucleotide is eliminated from each end of the viral DNA (Fig 1). The resulting cleaved DNA is then used as a substrate for integration or strand transfer leading to the covalent insertion of the viral DNA into the genome of the infected cell (Fig 1). This second reaction occurs simultaneously at both ends of the viral DNA molecule, with an offset of precisely five base pairs between the two opposite points of insertion.

These two reactions also occur *in vivo *in a sequential manner. The two reactions are also energetically independent. In both cases, the reaction is a single-step *trans*-esterification involving the disruption of a phosphodiester bond by nucleophilic attack. In the first reaction, the bond concerned is part of the viral DNA molecule and in the second, the bond is in the target DNA. There is therefore no covalent intermediate between the enzyme and the DNA as it is observed during catalytic reaction of topoisomerase or IN of lambda phage, for example. The removal of the dinucleotides from the 5' overhang, of viral origin, and DNA repair (*i.e. *polymerisation and ligation) are required to complete the full integration reaction. One study suggested that this might involve a DNA-dependent DNA polymerase activity of the IN [41], but, to date, such a polymerase activity of IN was not confirmed and it is generally thought that this DNA repair is performed by cellular mechanisms that can be reproduced *in vitro *with purified host cell factors [42]. The final reaction thus results in a viral DNA molecule, the provirus, integrated into and collinear with the genomic DNA, with a characteristic 5 base pairs duplication (in the case of HIV-1) of genomic sequence flanking the integration site. Several lines of evidence support a non-random integration with preferential integration in transcription units for HIV-1 [43]. Integration is then mainly directed by interactions between the pre-integration complex and chromatin. From a DNA sequence point of view, it was recently shown that integration occurs preferentially within symmetric sequences [44-46] (see # 2.3).

Both reactions (3'-processing and strand transfer) can be reproduced *in vitro *using short double-stranded oligonucleotides mimicking the sequence of the ends of the viral LTR U5 or U3 in the presence of a recombinant integrase [47]. 3'-processing is a highly specific reaction. This reaction involves the removal of a dinucleotide, adjacent to the highly conserved CA dinucleotide, from the 3' strand of the U3 and U5 viral DNA LTRs. Mutations in this sequence completely abolish activity, whereas the integrity of flanking sequences is much less important [15,48]. The 3'-processing reaction corresponds to a nucleophilic attack by a water molecule. However, other alternative nucleophilic agents can be used such as glycerol but generally conduct to non-specific endonucleolytic cleavage [49-51]. This mainly occurs when Mn^2+ ^is used. The 3'-OH of the unprocessed DNA can also be used directly as a nucleophilic agent leading to 3'-5' cyclic dinucleotide product [49]. The use of the physiological relevant cofactor Mg^2+ ^improves the specificity of the cleavage with water as the mainly used nucleophilic agent.

During the same reaction, IN can catalyse, with a modest yield, the strand transfer. In the strand transfer reaction, the nucleophilic agent corresponds to the 3'-OH extremity of the processed strand. It is possible to increase the yield of the strand transfer with pre-processed oligonucleotides [36]. By using an oligonucleotide mimicking one LTR end, only a half-transfer reaction can be observed. *In vitro*, long DNA fragments with two viral extremities can be used to reproduce the concerted integration process which corresponds to the simultaneous integration of two viral ends [35,36,52,53]. Concerted integration appears less tolerant to reaction conditions, *i.e*. enzyme preparation and oligomerization state than strand transfer. Although it was shown by different groups that IN alone is sufficient to catalyse the concerted integration, viral or cellular protein, acting as cofactors for the integration process, such as the viral nucleocapsid protein NC [54] and the cellular proteins HMG I(Y) [55] and LEDGF [56-58] may increase its efficacy. Interestingly, it was recently shown that, in contrast to the half-transfer reaction, a higher reaction yield was obtained for the concerted integration starting from a blunt-ended as compared to a pre-processed DNA substrate [36]. Furthermore, activity of IN is strongly dependent on its oligomeric state [14,47,59]. In contrast to 3'-processing which requires the dimeric form of IN [31], it was shown that concerted integration requires a tetrameric organization [32].

Both the 3'-processing and strand transfer reactions require a metallic cofactor. This cofactor may be Mn^2+ ^or Mg^2+^, but Mg^2+ ^is preferentially used *in vivo*. Indeed, there is considerable experimental evidence to suggest that Mg^2+ ^is more physiologically relevant, particularly as the specificity of the reaction is much greater in the presence of this cation: (i) IN displays strong non-specific nuclease activity in the presence of Mn^2+ ^[60,61]. (ii) The tolerance of sequence variation at the ends of the viral DNA molecule is much greater in the presence of Mn^2+ ^than in the presence of Mg^2+ ^[15,48]. (iii) Many IN mutations remain silent in the presence of Mn^2+ ^but not in the presence of Mg^2+^. For example, mutations of the HHCC domain that are deleterious to the virus *in vivo *affect 3'-processing and integration activities in *in vitro *tests using Mg^2+^, but have no such effect in tests using Mn^2+ ^[62,63]. Furthermore, zinc has no stimulatory effect on IN activity when using Mn^2+ ^as a cofactor while zinc stimulates the Mg^2+^-dependent activity [14]. In the Pearson Hard-Soft Acid-Base theory (HSBA), hards metal ions such as Mg^2+ ^(with d^0 ^electron configuration) are characterized by electron clouds which are not easily deformed, in contrast to soft metals ions such as Mn^2+^, with direct consequences on the active site plasticity and reaction specificity for many metal-dependent enzymes when comparing their activities under either Mg^2+ ^or Mn^2+ ^context. The presence of Mg^2+ ^generally leads to more stringent conditions for catalysis in term of reaction specificity as found for RAG1/2 proteins [64], Tn10 transposase [65], RNase H activity [66]. HIV-1 integrase also displays such a differential qualitative behaviour between Mg^2+ ^and Mn^2+^-dependent catalysis. It was also reported that IN/DNA complexes display different stabilities depending on the cofactor context with IN/DNA complexes being more stable in the presence of Mn^2+ ^than in the presence of Mg^2+ ^[67-69]. Such a differential stability of complexes is generally observed using IN purified in the presence of detergent and accounts for quantitative differences in term of enzymatic activity when comparing Mg^2+ ^and Mn^2+^. Indeed, IN from detergent-containing preparations displays more Mn^2+^-dependant than Mg^2+^-dependant activity as compared to detergent-free preparations that quantitatively display similar activities. The difference between cofactors has pharmacological implications, as the apparent efficacy of various IN inhibitors differs between tests using Mg^2+ ^or Mn^2+ ^as a cofactor [70-72], and the effects of mutations conferring drug resistance are often detectable only in tests using Mg^2+ ^as the cofactor [73]. These considerations have led to the use of chemical groups chelating Mg^2+ ^in the rational design of integrase inhibitors. Such groups are present in all the inhibitors developed to date, including raltegravir and elvitegravir [1].

**Figure 2:**
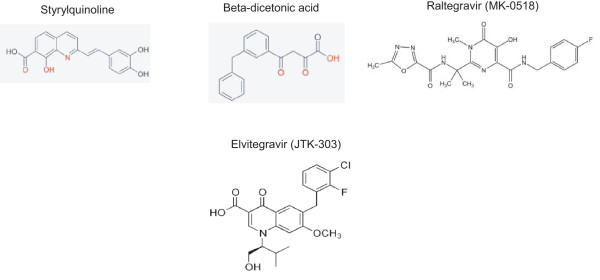
Some anti-integrase compounds. Styrylquinoline, a member of the INBI (IN DNA-Binding Inhibitors) compound and beta dicetonic acid, Raltegravir and Elvitegravir, members of the INSTI (IN Strand Transfer Inhibitors) compounds, are represented.

Whatever the activity tested, IN is characterized by an overall slow cleavage efficiency. Furthermore, IN form stable complexes with both DNA substrate and DNA product, limiting multiple turnover [74]. Taken together, these features resemble to those observed for other polynucleotidyl-tranferases such as transposases. These enzymes share a peculiar enzymatic property: they have evolved to catalyse multi-sequential steps (two reactions for IN and four for Tn5 transposase) in a single active site. A multi-sequential reaction requires a strong binding of the enzyme to the DNA product after each chemical step to optimise the entire process but consequently diminishes the overall enzymatic efficacy in term of turnover. However, this weak catalytic activity is not detrimental for these enzymes in the cellular context, because a single event of integration or transposition is sufficient for the overall function. *In vivo*, this tight binding of IN to the viral processed DNA most likely allows the complex to remain associated after the 3'-processing reaction long enough for subsequent integration. Two strategies have been considered for the development of IN inhibitors: screening using the unbound protein (before complex formation) or screening with the preformed IN-viral DNA complex. The success of these two approaches has been demonstrated by the identification of (i) inhibitors of 3'-processing targeting the DNA free enzyme and blocking its binding to the viral DNA [75] and (ii) inhibitors of strand transfer targeting the preformed complex more related to the preintegration complex (PIC) [76]. These two families of compounds are respectively called INBI (IN DNA-Binding Inhibitors) and INSTI (IN Strand Transfer Inhibitors) (Fig 2). Since the early 1990s, a number of compounds inhibiting either 3'processing or strand transfer have been identified *in vitro *[77,78]. The great stability of the PIC and its presence in the cell throughout most of the preintegration steps make this complex the most suitable target. Unfortunately, most of the INBI compounds are inactive on the preformed complexes. Indeed only strand transfer inhibitors or INSTIs have been shown to be potent antiviral compounds. As they selectively target the preformed IN-viral DNA complex and inhibit the binding of the acceptor DNA (*i.e. *target DNA or host DNA), INSTI compounds selectively inhibit the strand transfer reaction and have no effect on the 3'-processing reaction [79]. One such compound, Raltegravir (Isentress@), which was developed based on early studies by Hazuda et al. [76], was approved for clinical use in Autumn 2007 as the first antiretroviral drug (ARV) targeting the viral integrase (IN). This inhibitor act by binding to the IN-viral DNA complex, close to the 3' end of the donor DNA, thereby selectively blocking the strand transfer step; the IC_50 _values are in the nanomolar range both *in vitro *and *ex vivo *with a high therapeutic index [80]. Unfortunately, variants of the virus resistant to this inhibitor have already been reported [80]. The emergence of resistant virus *in vivo *should prompt both a search for new INSTIs and reassessment of the potential inhibitory activity of INBIs (such as styrylquinolines or SQL) which have been shown to be inhibitors of 3'processing *in vitro *with significant inhibitory activity against viral replication in cell cultures (Fig 2) [81].

The presence either of the catechol or an another group on the SQLs able to form a complex of coordination with a divalent ion suggests that these compounds interact with the active site of the enzyme by a chelation with the metallic cofactor. These compounds are mainly inhibitors of the 3'-processing reaction, and their mechanism of action *in vitro *can be assimilated to a competitive mechanism. Recently, experiments based on fluorescence anisotropy demonstrated that SQLs are DNA-binding inhibitors of HIV-1 IN [75]. In summary, INBI compounds primarily compete with the binding of the donor DNA (viral DNA) while INSTI compounds compete with the binding of the acceptor DNA (target DNA). However, the mechanism of inhibition of SQLs in the cell context is not completely understood. These compounds appear to act at steps prior to integration, more particularly during RT [82] and nuclear import [83]. These effects are mediated by IN as evidenced by the appearance of resistance mutation in IN sequence. It is then suggested that, *ex vivo*, non catalytic region of IN are targeted by SQs (see paragraph "non catalytic role of IN"). It is interesting to note that the two classes of IN inhibitors, INBI and INSTI, induce distinct resistant mutations [76,82,84-86].

#### Disintegration

A third reaction, disintegration, is observed *in vitro *(Fig 1). Disintegration may be considered to be the reverse of the strand transfer reaction [2]. Unlike the 3'-processing and strand transfer reactions which requires the full-length protein, the disintegration reaction can be catalysed by the catalytic domain alone (IN^55–212^) or by truncated proteins, IN^1–212 ^or IN^55–288 ^[47,87,88]. This activity was widely used for testing the competitive mechanisms of certain inhibitors. There is currently no experimental evidence to suggest that this reaction occurs *in vivo*.

#### A new internal specific activity

Recently, our group has identified a new internal and specific cleavage activity *in vitro *of HIV-1 IN [3]. Until now, all attempts to study a specific internal endonucleolytic cleavage *in vitro *have failed. Vink et *al. *have demonstrated that when the CA dinucleotide, indispensable for the 3'-processing, was separated by more than 2 nucleotides from the 3'-OH end, the activity was dramatically impaired [89]. Nevertheless, we have demonstrated that oligonucleotides mimicking the palindromic sequence found at the LTR-LTR junction of the 2-LTR circles (found in infected cells) were efficiently cleaved at internal positions by HIV-1 IN, with cleavage kinetics comparable to the 3'-processing reaction (Fig 1). This reaction occurs symmetrically on both strands, with a strong cleavage at the CA dinucleotide (corresponding to the CA sequence used for the 3'-processing reaction). A second weaker cleavage site appears after the next adenine (TA sequence) in the 5'-3' direction. Furthermore, HIV-1 IN can efficiently cleave a plasmid mimicking the 2-LTR circles specifically at the LTR-LTR junction. The specificity of this reaction is similar to the one catalysed by transposases which cleave the DNA substrate after a CA or TA dinucleotide [90]. Such internal cleavages are not observed using a mutant of the catalytic site (E152A) testifying that the DDE triad is also implicated in this reaction. In addition, this novel activity is stringent and highly specific as (i) it occurs with the physiological metallic cofactor (Mg^2+^) and not only Mn^2+^, (ii) only the full-length IN is competent for the internal cleavage of the palindrome, in contrast to the disintegration reaction that is efficiently catalysed by truncated proteins such as IN^55–212^, IN^55–288^, IN^1–212 ^and (iii) it does not sustain any mutation in the sequence of the LTR-LTR junction. Furthermore, the cleavage of the LTR-LTR junction requires the tetrameric forms of IN whereas the 3'-processing reaction is efficiently catalysed by a dimer [31,32]. This new activity seems to be generalised to other retroviral IN as reported earlier for PFV-1 IN [91,92]. However, although PFV-1 IN performs this cleavage activity, it is important to note that both IN are strictly restricted to their own cognate palindromic sequence: HIV-1 IN is unable to cleave the PFV LTR-LTR junction and PFV-1 IN is unable to cleave the HIV-1 LTR-LTR junction.

Recently, mapping of extensive integration sites, notably for HIV-1, put in light the existence of a weak palindromic consensus [44-46]. It is important to note that the sequence of the weak palindromic consensus is similar, although not identical, to the one found at the LTR-LTR junction. This specific endonucleolytic activity on a palindromic LTR-LTR junction as well as the symmetrical organization of integration sites reveal a common structural feature of IN: IN intrinsically prefers to bind to symmetric DNA sequences. Moreover, we have found that tetramers catalyses the cleavage of the palindromic sequence while others have suggested that the same oligomeric form is responsible for the concerted integration in the context of the synaptic complex [35,36]. Therefore, one could reasonably imagine that the same multimeric organization of IN (*i.e. *the tetrameric form) is stabilised by a corresponding symmetry at the DNA level, either at the viral DNA (LTR-LTR junction) or at the target level (integration sites).

*In vivo*, unintegrated viral DNA could represent 99% of total viral DNA in infected cells [93] underlying that integration is a rare event. Un-integrated DNA is mainly linear but also circular – 1-LTR or 2-LTR circles. In the absence of integration (for example using strand-transfer inhibitors such as diketo acids), at least the 2-LTR circular forms of viral DNA, which are usually believed to be dead-end molecules, are accumulated [94]. It is tempting to speculate about a possible role of 2-LTR circles in a subsequent integration process after removing the drug pressure, mediated by the ability of IN to cleave the LTR-LTR junction. However, to date, although IN is able to cleave the LTR-LTR junction *in vitro*, there is no proof that such a cleavage can occur *in vivo *and thus that 2-LTR circles could be an efficient precursor for integration.

#### Modulation of IN activity

Several cellular and viral proteins have been reported to stimulate IN activities *in vitro *as well as *in vivo*. Among these cofactors, some proteins are known to interact directly with IN and thus enhance its solubility or favours an active conformation of IN, while other proteins do not physically interact with IN but could indirectly stimulate IN activities as found for proteins playing a structural role on DNA conformation.

For instance, in the group of IN interactors, the yeast chaperoning protein, yHSP60, was described by Parissi and colleagues to interact directly with HIV-1 IN [95]. It has also been demonstrated that the human counterpart of the yHSP60, hHSP60, was able to stimulate the *in vitro *processing as well as joining activities of IN, suggesting that hHSP60-IN interaction could allow IN to adopt a more competent conformation for activity or prevent IN from aggregation [95]. However, further investigations must be done to confirm the potential role of HSP60 in the viral life cycle.

LEDGF/p75, Lens Epithelial Derived Growth Factor, has been reported to interact with IN and stimulate both concerted integration and strand transfer. Addition of recombinant LEDGF/p75 to an *in vitro *mini HIV-based IN assay enhanced the strand transfer activity of the recombinant HIV-1 IN [56]. This stimulation is highly dependent of the ratio between IN and LEDGF used for the reaction [58]. Probably, LEDGF/p75 has a double effect on IN. The first one is similar to the one described for HSP60. Indeed, it was shown that LEDGF-IN complex displays a more favourable solubility profiles as compared to the free IN [96]. In the same publication, a second effect could explain the enhancement of IN activity as LEDGF/p75 binding to DNA concomitantly increases IN-DNA affinity [96]. Concerning more specifically the concerted integration, it has been reported that LEDGF increases the stabilisation of the tetrameric state of IN which is responsible for the concerted integration [97]. In vivo, LEDGF displays an important role in the targeting of the viral integration [98] (see also # 2.5).

It is important to note that IN activity is also highly regulated by the structure of the viral and host DNA substrates which can be influenced by protein interactions on DNA. Pruss *et al. *studied the propensity of IN to integrate an oligonucleotide mimicking the HIV LTR into either DNA molecules of known structure or nucleosomal complexes [99,100]. Results highlight that the structure of the target greatly influences the site of integration, and that DNA curvature, flexibility/rigidity in solution, all parameters influence the frequency of integration. Furthermore, using a model target comprising a 13-nucleosome extended array that includes binding sites for specific transcription factors and which can be compacted into a higher-ordered structure, Taganov *et al*. demonstrated that the efficiency of the *in vitro *integration was decreased after compaction of this target with histone H1 [101]. Consequently, both intrinsic DNA structure and the folding of DNA into chromosomal structures will exert a major influence on both catalysis efficiency and target site selection for the viral genome integration. The structure of the viral DNA also greatly influences IN activity [102], as illustrated by alterations in the minor groove of the viral DNA which result in a greater decrease in 3'-processing activity than major groove substitutions, suggesting a great importance of the structure of the viral DNA for IN activities.

Several cellular proteins greatly influence the structure of the viral DNA and thus modulate IN activities. For example, BAF (Barrier-to-autointegration factor), a component of the functional HIV-1 pre-integration complex, stimulates the integration reaction in the PIC complex [103,104]. The effect of BAF on integration is probably due, *in vitro*, to its DNA binding activity and its effect on the viral DNA structure [105]. HMG I(Y), a protein partner of the HIV-1 PICs, has been also described to stimulate concerted integration *in vitro*. Li and colleagues demonstrated that HMG I(Y) can condense model HIV-1 cDNA *in vitro*, possibly by approximating both LTR ends and facilitating IN binding by unwinding the LTR termini [106]. These data suggest that binding of HMG I(Y) to multiple cDNA sites compacts retroviral cDNA, thereby promoting formation of active integrase-cDNA complexes [106]. In addition, Carteau and colleagues led to the finding that concerted integration can be stimulated more than 1,000-fold in the presence of the nucleocapsid protein in comparison to integrase alone under some conditions of reaction [54]. To date, the effect of the NC on concerted integration is not clear but is probably due its capability to promote DNA distorsion.

Another IN cofactor, INI-1 (Integrase Interactor 1), has been described to enhance IN activity probably by structural and topological effect on DNA. INI-1, is one of the core subunits of the ATP-dependent chromatin remodelling complex SWI/SNF that regulates expression of numerous eukaryotic genes by altering DNA/histone interaction. INI-1 was identified by a two-hybrid system that binds to IN and enhances the strand transfer activity of the protein [107]. Taking into account that INI-1 interacts with IN, it is not excluded that a solubility effect induced by protein-protein interaction may account for the stimulation effect on IN activity as reported for LEDGF/p75. It is important to note that conflicting results concerning the role of INI-1 in the HIV-1 life cycle have been reported. It has been described that SNF5/INI-1 interferes with early steps of HIV-1 replication [108]. Boese and colleagues found no effects on viral integration in cells depleted for INI-1 [109], whereas it has been proposed that INI-1 was required for efficient activation of Tat-mediated transcription [110]. The comprehension of the role of such IN partners, as well as the discovery of novel partners will be crucial to reproduce more authentic integrase complexes for mechanistic studies and development of IN inhibitors.

#### Targeting viral integration

Additionally, interactions between IN and cellular protein partners play key role in the targeting of integration. A systematic study of the sites of HIV DNA integration into the host DNA has shown that integration is not entirely random. Analysis of integration sites *in vivo *indicates that HIV tends to integrate into sites of active transcription [43]. It is likely that this integration bias results from interactions between PICs and components of cellular origin in relationship with the chromatin tethering. Several cellular cofactors, including INI-1 [107,111], BAF [103,112], Ku [113] and LEDGF/p75 [114], are known to interact with the PIC in the nucleus. Among these proteins, at least INI-1 and LEDGF/p75 physically interact with IN [107,115]. Recent work with LEDGF/p75 strongly suggests that this cofactor is actually responsible for targeting integration [11]. LEDGF/p75 silencing modifies the bias from transcription units to CpG islands [43,116]. As LEDGF/p75 is essential for HIV-1 replication and LEDGF/p75 interacts directly with IN, the domain of interaction between these two proteins is therefore a promising target for the development of integrase ligands with antiviral activity. Although no direct interaction between IN and BAF or Ku was described, it is suggested that these two cofactors could influence the profile or efficiency of integration [117,118]. For example, interaction of BAF with emerin, an internal-inner-nuclear-envelope protein, could favour the access of the PIC to the chromatin and thus facilitate integration [119]. In relationship with chromatin, it was recently described that the C-terminal domain of IN is acetylated by a histone acetyl transferase (HAT) [120]. However, the effect of IN acetylation on integration *in vivo *remains unclear [121].

### Non catalytic activities of IN

IN plays a key role for retroviral replication because of its catalytical activities. In addition, IN has also non catalytic properties that are essential for the replication cycle. Mutations of IN can be divided into two groups. The first class of mutations (Class I) includes mutants that are affected in their catalytic activities. For instance, one mutation in either the three amino acids of the DDE triad abolishes the catalytic activities of IN. The second class of mutations (Class II mutants) disturbs other steps of the retroviral replication and corresponding purified integrase mutants display wild-type level of activity.

Several mutations of IN displayed an *in vivo *DNA synthesis defect and a block of viral replication at the reverse transcription level [8,9,122-124]. A structural general defect at the level of the retrotranscription complex which includes RT and IN may account for such a phenotype. Indeed, several studies suggest a physical interaction between IN and RT [9]. Such a defect in DNA synthesis can be also observed when using SQL compounds which target integrase, as evidenced by resistance mutations study, but primarily affect the reverse transcription step [82].

Another role of IN prior to integration is related to the PIC translocation in the nucleus. In fact, in non-dividing infected cells, such as macrophages, the PIC must cross the nuclear membrane to reach the chromosomal DNA. This involves an active mechanism, the determinants of which remain unclear [125,126]. It has been reported by De Soultrait *et al. *that L2, which corresponds to the C-end half of the yeast STU2p, a microtubule-associated protein (MAP), interacts with IN. STU2p is an essential component of the yeast spindle pole body (SPB), which is able to bind microtubules in vitro. This interaction was observed *in vitro *and also *in vivo *in a yeast model [127]. The identification of components of the microtubule network associated with IN suggests a role of this complex in the transport of HIV-1 PIC to the nucleus and supports recent particle tracking data suggesting that PIC is characterized by a microtubule-directed movement [128].

Integrase and at least two other components of the PIC, Vpr and MA, have karyophilic properties [129] suggesting that several distinct mechanisms could be involved in the nuclear import. The integrase enzyme includes several sequence motifs likely to act as nuclear localisation signals (NLSs), including at least one known to interact with the nuclear import receptor, this motif being located in the C-terminal domain [126]. A sequence within the catalytic core including the V165 and R166 residues may also contribute to the karyophilic properties of integrase [130], although this remains a matter of debate [124,131]. In any case, the mutation of these various sequences does not completely abolish the nuclear translocation of PICs, confirming that there are complementary and/or redundant translocation mechanisms. Recently, a novel partner of IN in the nuclear translocation has been described by Christ and colleagues [132]. Using yeast two-hybrid and pull-down experiments, the transportin-SR2 (TRN-SR2) was shown to interact with IN. By RNAi experiment on infected cells, SR2 was clearly validated as an essential partner in the translocation of IN and consequently of the PIC into the nucleus of infected cells.

Finally, integrase could be indirectly involved in the regulation of transcription of integrated provirus. After the integration process, IN could be tightly bound to the integrated DNA and then, the degradation of IN by the proteasome-ubiquitin pathway was proposed to regulate the transcription of viral genes. Indeed, Dargemont and collaborators have found that integrase interacts with VBP1 (von Hippel-Lindau binding protein 1), a binding partner of Cul2/VHL ligase complex involved in the polyubiquitylation process [133].

## Conclusion

In conclusion, remarkable progress has been made towards understanding the structure of the pre-integration complex formed by HIV integrase and viral DNA. This new knowledge has led to considerable improvements in the methods used to search for compounds active against this enzyme. Several families of inhibitors have now been identified, including at least one – strand transfer inhibitors – currently in the advanced stages of clinical development and giving results sufficiently promising for one molecule (Raltegravir) to have obtained a licence in October 2007 for release in the United States. The identification of several new integrase cofactors will provide us with a clearer picture of the determinants of integration *in vivo*, opening up new possibilities for pharmacological research [134]. There is no doubt that interest in the structural biology of integrase will be substantially stimulated by the release of the first integrase inhibitors onto the market and, unfortunately, by the likely emergence of resistant viruses.

## Abbreviations

HIV-1: Human Immunodeficiency virus type 1; PFV-1: Primate Foamy virus type 1; MK-0518: Raltegravir; ARVs: Antiretroviral drugs; IN: Integrase; RT: Reverse Transcriptase; MA: Matrix; NC: Nucleocapsid; LTR: Long Terminal Repeat; INBI: IN DNA-Binding Inhibitor; INSTI: IN Strand Transfer Inhibitor; PIC: Pre-integration Complex; LEDGF: Lens Epithelial Derived Growth Factor

## Competing interests

The authors declare that they have no competing interests.

## Authors' contributions

OD and JFM are the principal investigators. OD, KC, AS, ED and JFM wrote the manuscript. All authors read and approved the manuscript.

## References

[B1] Al Mawsawi LQ, Al Safi RI, Neamati N (2008). Anti-infectives clinical progress of HIV-1 integrase inhibitors. Expert Opin Emerg Drugs.

[B2] Chow SA, Vincent KA, Ellison V, Brown PO (1992). Reversal of integration and DNA splicing mediated by integrase of human immunodeficiency virus. Science.

[B3] Delelis O, Parissi V, Leh H, Mbemba G, Petit C, Sonigo P, Deprez E, Mouscadet JF (2007). Efficient and specific internal cleavage of a retroviral palindromic DNA sequence by tetrameric HIV-1 integrase. PLoS ONE.

[B4] Miller MD, Farnet CM, Bushman FD (1997). Human immunodeficiency virus type 1 preintegration complexes: studies of organization and composition. J Virol.

[B5] Bukrinsky MI, Sharova N, McDonald TL, Pushkarskaya T, Tarpley WG, Stevenson M (1993). Association of integrase, matrix, and reverse transcriptase antigens of human immunodeficiency virus type 1 with viral nucleic acids following acute infection. Proc Natl Acad Sci USA.

[B6] Nermut MV, Fassati A (2003). Structural analyses of purified human immunodeficiency virus type 1 intracellular reverse transcription complexes. J Virol.

[B7] Gallay P, Swingler S, Song J, Bushman F, Trono D (1995). HIV nuclear import is governed by the phosphotyrosine-mediated binding of matrix to the core domain of integrase. Cell.

[B8] Wu X, Liu H, Xiao H, Conway JA, Hehl E, Kalpana GV, Prasad V, Kappes JC (1999). Human immunodeficiency virus type 1 integrase protein promotes reverse transcription through specific interactions with the nucleoprotein reverse transcription complex. J Virol.

[B9] Zhu K, Dobard C, Chow SA (2004). Requirement for integrase during reverse transcription of human immunodeficiency virus type 1 and the effect of cysteine mutations of integrase on its interactions with reverse transcriptase. J Virol.

[B10] Dobard CW, Briones MS, Chow SA (2007). Molecular mechanisms by which human immunodeficiency virus type 1 integrase stimulates the early steps of reverse transcription. J Virol.

[B11] Llano M, Saenz DT, Meehan A, Wongthida P, Peretz M, Walker WH, Teo W, Poeschla EM (2006). An Essential Role for LEDGF/p75 in HIV Integration. Science.

[B12] Hombrouck A, De Rijck J, Hendrix J, Vandekerckhove L, Voet A, De Maeyer M, Witvrouw M, Engelborghs Y, Christ F, Gijsbers R (2007). Virus evolution reveals an exclusive role for LEDGF/p75 in chromosomal tethering of HIV. PLoS Pathog.

[B13] Zheng R, Jenkins TM, Craigie R (1996). Zinc folds the N-terminal domain of HIV-1 integrase, promotes multimerization, and enhances catalytic activity. Proc Natl Acad Sci USA.

[B14] Lee SP, Xiao J, Knutson JR, Lewis MS, Han MK (1997). Zn2+ promotes the self-association of human immunodeficiency virus type-1 integrase in vitro. Biochemistry.

[B15] Esposito D, Craigie R (1998). Sequence specificity of viral end DNA binding by HIV-1 integrase reveals critical regions for protein-DNA interaction. EMBO J.

[B16] Jenkins TM, Esposito D, Engelman A, Craigie R (1997). Critical contacts between HIV-1 integrase and viral DNA identified by structure-based analysis and photo-crosslinking. EMBO J.

[B17] Heuer TS, Brown PO (1997). Mapping features of HIV-1 integrase near selected sites on viral and target DNA molecules in an active enzyme-DNA complex by photo-cross-linking. Biochemistry.

[B18] Drake RR, Neamati N, Hong H, Pilon AA, Sunthankar P, Hume SD, Milne GW, Pommier Y (1998). Identification of a nucleotide binding site in HIV-1 integrase. Proc Natl Acad Sci USA.

[B19] Johnson AA, Santos W, Pais GC, Marchand C, Amin R, Burke TR, Verdine G, Pommier Y (2006). Integration requires a specific interaction of the donor DNA terminal 5'-cytosine with glutamine 148 of the HIV-1 integrase flexible loop. J Biol Chem.

[B20] Goldgur Y, Dyda F, Hickman AB, Jenkins TM, Craigie R, Davies DR (1998). Three new structures of the core domain of HIV-1 integrase: an active site that binds magnesium. Proc Natl Acad Sci USA.

[B21] Maignan S, Guilloteau JP, Zhou-Liu Q, Clement-Mella C, Mikol V (1998). Crystal structures of the catalytic domain of HIV-1 integrase free and complexed with its metal cofactor: high level of similarity of the active site with other viral integrases. J Mol Biol.

[B22] Cai M, Zheng R, Caffrey M, Craigie R, Clore GM, Gronenborn AM (1997). Solution structure of the N-terminal zinc binding domain of HIV-1 integrase. Nat Struct Biol.

[B23] Lodi PJ, Ernst JA, Kuszewski J, Hickman AB, Engelman A, Craigie R, Clore GM, Gronenborn AM (1995). Solution structure of the DNA binding domain of HIV-1 integrase. Biochemistry.

[B24] Wang JY, Ling H, Yang W, Craigie R (2001). Structure of a two-domain fragment of HIV-1 integrase: implications for domain organization in the intact protein. EMBO J.

[B25] Chen JC, Krucinski J, Miercke LJ, Finer-Moore JS, Tang AH, Leavitt AD, Stroud RM (2000). Crystal structure of the HIV-1 integrase catalytic core and C-terminal domains: a model for viral DNA binding. Proc Natl Acad Sci USA.

[B26] van Gent DC, Vink C, Groeneger AA, Plasterk RH (1993). Complementation between HIV integrase proteins mutated in different domains. EMBO J.

[B27] Engelman A, Bushman FD, Craigie R (1993). Identification of discrete functional domains of HIV-1 integrase and their organization within an active multimeric complex. EMBO J.

[B28] Ent FM van den, Vos A, Plasterk RH (1999). Dissecting the role of the N-terminal domain of human immunodeficiency virus integrase by trans-complementation analysis. J Virol.

[B29] Fletcher TM, Soares MA, McPhearson S, Hui H, Wiskerchen M, Muesing MA, Shaw GM, Leavitt AD, Boeke JD, Hahn BH (1997). Complementation of integrase function in HIV-1 virions. EMBO J.

[B30] Deprez E, Tauc P, Leh H, Mouscadet JF, Auclair C, Hawkins ME, Brochon JC (2001). DNA binding induces dissociation of the multimeric form of HIV-1 integrase: a time-resolved fluorescence anisotropy study. Proc Natl Acad Sci USA.

[B31] Guiot E, Carayon K, Delelis O, Simon F, Tauc P, Zubin E, Gottikh M, Mouscadet JF, Brochon JC, Deprez E (2006). Relationship between the oligomeric status of HIV-1 integrase on DNA and enzymatic activity. J Biol Chem.

[B32] Faure A, Calmels C, Desjobert C, Castroviejo M, Caumont-Sarcos A, Tarrago-Litvak L, Litvak S, Parissi V (2005). HIV-1 integrase crosslinked oligomers are active in vitro. Nucleic Acids Res.

[B33] Baranova S, Tuzikov FV, Zakharova OD, Tuzikova NA, Calmels C, Litvak S, Tarrago-Litvak L, Parissi V, Nevinsky GA (2007). Small-angle X-ray characterization of the nucleoprotein complexes resulting from DNA-induced oligomerization of HIV-1 integrase. Nucleic Acids Res.

[B34] Delelis O, Carayon K, Guiot E, Leh H, Tauc P, Brochon JC, Mouscadet JF, Deprez E (2008). Insight into the integrase-DNA recognition mechanism. A specific DNA-binding mode revealed by an enzymatically labeled integrase. J Biol Chem.

[B35] Li M, Mizuuchi M, Burke TR, Craigie R (2006). Retroviral DNA integration: reaction pathway and critical intermediates. EMBO J.

[B36] Li M, Craigie R (2005). Processing of viral DNA ends channels the HIV-1 integration reaction to concerted integration. J Biol Chem.

[B37] Gao K, Butler SL, Bushman F (2001). Human immunodeficiency virus type 1 integrase: arrangement of protein domains in active cDNA complexes. EMBO J.

[B38] Podtelezhnikov AA, Gao K, Bushman FD, McCammon JA (2003). Modeling HIV-1 integrase complexes based on their hydrodynamic properties. Biopolymers.

[B39] Ren G, Gao K, Bushman FD, Yeager M (2007). Single-particle image reconstruction of a tetramer of HIV integrase bound to DNA. J Mol Biol.

[B40] Wielens J, Crosby IT, Chalmers DK (2005). A three-dimensional model of the human immunodeficiency virus type 1 integration complex. J Comput Aided Mol Des.

[B41] Acel A, Udashkin BE, Wainberg MA, Faust EA (1998). Efficient gap repair catalyzed in vitro by an intrinsic DNA polymerase activity of human immunodeficiency virus type 1 integrase. J Virol.

[B42] Brin E, Yi J, Skalka AM, Leis J (2000). Modeling the late steps in HIV-1 retroviral integrase-catalyzed DNA integration. J Biol Chem.

[B43] Marshall HM, Ronen K, Berry C, Llano M, Sutherland H, Saenz D, Bickmore W, Poeschla E, Bushman FD (2007). Role of PSIP1/LEDGF/p75 in lentiviral infectivity and integration targeting. PLoS ONE.

[B44] Grandgenett DP (2005). Symmetrical recognition of cellular DNA target sequences during retroviral integration. Proc Natl Acad Sci USA.

[B45] Holman AG, Coffin JM (2005). Symmetrical base preferences surrounding HIV-1, avian sarcoma/leukosis virus, and murine leukemia virus integration sites. Proc Natl Acad Sci USA.

[B46] Wu X, Li Y, Crise B, Burgess SM, Munroe DJ (2005). Weak palindromic consensus sequences are a common feature found at the integration target sites of many retroviruses. J Virol.

[B47] Leh H, Brodin P, Bischerour J, Deprez E, Tauc P, Brochon JC, LeCam E, Coulaud D, Auclair C, Mouscadet JF (2000). Determinants of Mg2+-dependent activities of recombinant human immunodeficiency virus type 1 integrase. Biochemistry.

[B48] Agapkina J, Smolov M, Barbe S, Zubin E, Zatsepin T, Deprez E, Le Bret M, Mouscadet JF, Gottikh M (2006). Probing of HIV-1 integrase/DNA interactions using novel analogs of viral DNA. J Biol Chem.

[B49] Engelman A, Mizuuchi K, Craigie R (1991). HIV-1 DNA integration: mechanism of viral DNA cleavage and DNA strand transfer. Cell.

[B50] Gerton JL, Herschlag D, Brown PO (1999). Stereospecificity of reactions catalyzed by HIV-1 integrase. J Biol Chem.

[B51] Skinner LM, Sudol M, Harper AL, Katzman M (2001). Nucleophile selection for the endonuclease activities of human, ovine, and avian retroviral integrases. J Biol Chem.

[B52] Sinha S, Pursley MH, Grandgenett DP (2002). Efficient concerted integration by recombinant human immunodeficiency virus type 1 integrase without cellular or viral cofactors. J Virol.

[B53] Sinha S, Grandgenett DP (2005). Recombinant human immunodeficiency virus type 1 integrase exhibits a capacity for full-site integration in vitro that is comparable to that of purified preintegration complexes from virus-infected cells. J Virol.

[B54] Carteau S, Gorelick RJ, Bushman FD (1999). Coupled integration of human immunodeficiency virus type 1 cDNA ends by purified integrase in vitro: stimulation by the viral nucleocapsid protein. J Virol.

[B55] Hindmarsh P, Ridky T, Reeves R, Andrake M, Skalka AM, Leis J (1999). HMG protein family members stimulate human immunodeficiency virus type 1 and avian sarcoma virus concerted DNA integration in vitro. J Virol.

[B56] Cherepanov P, Maertens G, Proost P, Devreese B, Van Beeumen J, Engelborghs Y, De Clercq E, Debyser Z (2003). HIV-1 integrase forms stable tetramers and associates with LEDGF/p75 protein in human cells. J Biol Chem.

[B57] Cherepanov P (2007). LEDGF/p75 interacts with divergent lentiviral integrases and modulates their enzymatic activity in vitro. Nucleic Acids Res.

[B58] Pandey KK, Sinha S, Grandgenett DP (2007). Transcriptional co-activator LEDGF/p75 modulates HIV-1 integrase mediated concerted integration. J Virol.

[B59] Deprez E, Tauc P, Leh H, Mouscadet JF, Auclair C, Brochon JC (2000). Oligomeric states of the HIV-1 integrase as measured by time-resolved fluorescence anisotropy. Biochemistry.

[B60] Engelman A, Craigie R (1995). Efficient magnesium-dependent human immunodeficiency virus type 1 integrase activity. J Virol.

[B61] Bushman FD, Wang B (1994). Rous sarcoma virus integrase protein: mapping functions for catalysis and substrate binding. J Virol.

[B62] Wiskerchen M, Muesing MA (1995). Human immunodeficiency virus type 1 integrase: effects of mutations on viral ability to integrate, direct viral gene expression from unintegrated viral DNA templates, and sustain viral propagation in primary cells. J Virol.

[B63] Khan E, Mack JP, Katz RA, Kulkosky J, Skalka AM (1991). Retroviral integrase domains: DNA binding and the recognition of LTR sequences. Nucleic Acids Res.

[B64] Hiom K, Gellert M (1997). A stable RAG1-RAG2-DNA complex that is active in V(D)J cleavage. Cell.

[B65] Junop MS, Haniford DB (1997). Factors responsible for target site selection in Tn10 transposition: a role for the DDE motif in target DNA capture. EMBO J.

[B66] Blain SW, Goff SP (1996). Differential effects of Moloney murine leukemia virus reverse transcriptase mutations on RNase H activity in Mg2+ and Mn2+. J Biol Chem.

[B67] Pemberton IK, Buc H, Buckle M (1998). Displacement of viral DNA termini from stable HIV-1 integrase nucleoprotein complexes induced by secondary DNA-binding interactions. Biochemistry.

[B68] Pemberton IK, Buckle M, Buc H (1996). The metal ion-induced cooperative binding of HIV-1 integrase to DNA exhibits a marked preference for Mn(II) rather than Mg(II). J Biol Chem.

[B69] Lesbats P, Metifiot M, Calmels C, Baranova S, Nevinsky G, Andreola ML, Parissi V (2008). In vitro initial attachment of HIV-1 integrase to viral ends: control of the DNA specific interaction by the oligomerization state. Nucleic Acids Res.

[B70] Fesen MR, Pommier Y, Leteurtre F, Hiroguchi S, Yung J, Kohn KW (1994). Inhibition of HIV-1 integrase by flavones, caffeic acid phenethyl ester (CAPE) and related compounds. Biochem Pharmacol.

[B71] Mazumder A, Neamati N, Ojwang JO, Sunder S, Rando RF, Pommier Y (1996). Inhibition of the human immunodeficiency virus type 1 integrase by guanosine quartet structures. Biochemistry.

[B72] Molteni V, Rhodes D, Rubins K, Hansen M, Bushman FD, Siegel JS (2000). A new class of HIV-1 integrase inhibitors: the 3,3,3', 3'-tetramethyl-1,1'-spirobi(indan)-5,5',6,6'-tetrol family. J Med Chem.

[B73] Grobler JA, Stillmock K, Hu B, Witmer M, Felock P, Espeseth AS, Wolfe A, Egbertson M, Bourgeois M, Melamed J (2002). Diketo acid inhibitor mechanism and HIV-1 integrase: implications for metal binding in the active site of phosphotransferase enzymes. Proc Natl Acad Sci USA.

[B74] Smolov M, Gottikh M, Tashlitskii V, Korolev S, Demidyuk I, Brochon JC, Mouscadet JF, Deprez E (2006). Kinetic study of the HIV-1 DNA 3'-end processing. FEBS J.

[B75] Deprez E, Barbe S, Kolaski M, Leh H, Zouhiri F, Auclair C, Brochon JC, Le Bret M, Mouscadet JF (2004). Mechanism of HIV-1 integrase inhibition by styrylquinoline derivatives in vitro. Mol Pharmacol.

[B76] Hazuda DJ, Felock P, Witmer M, Wolfe A, Stillmock K, Grobler JA, Espeseth A, Gabryelski L, Schleif W, Blau C (2000). Inhibitors of strand transfer that prevent integration and inhibit HIV-1 replication in cells. Science.

[B77] Semenova EA, Marchand C, Pommier Y (2008). HIV-1 integrase inhibitors: update and perspectives. Adv Pharmacol.

[B78] Egbertson MS (2007). HIV integrase inhibitors: from diketoacids to heterocyclic templates: a history of HIV integrase medicinal chemistry at Merck West Point and Merck Rome (IRBM). Curr Top Med Chem.

[B79] Espeseth AS, Felock P, Wolfe A, Witmer M, Grobler J, Anthony N, Egbertson M, Melamed JY, Young S, Hamill T (2000). HIV-1 integrase inhibitors that compete with the target DNA substrate define a unique strand transfer conformation for integrase. Proc Natl Acad Sci USA.

[B80] Malet I, Delelis O, Valantin MA, Montes B, Soulie C, Wirden M, Tchertanov L, Peytavin G, Reynes J, Mouscadet JF (2008). Mutations associated with failure of raltegravir treatment affect integrase sensitivity to the inhibitor in vitro. Antimicrob Agents Chemother.

[B81] Zouhiri F, Mouscadet JF, Mekouar K, Desmaele D, Savoure D, Leh H, Subra F, Le Bret M, Auclair C, d'Angelo J (2000). Structure-activity relationships and binding mode of styrylquinolines as potent inhibitors of HIV-1 integrase and replication of HIV-1 in cell culture. J Med Chem.

[B82] Bonnenfant S, Thomas CM, Vita C, Subra F, Deprez E, Zouhiri F, Desmaele D, d'Angelo J, Mouscadet JF, Leh H (2004). Styrylquinolines, integrase inhibitors acting prior to integration: a new mechanism of action for anti-integrase agents. J Virol.

[B83] Mousnier A, Leh H, Mouscadet JF, Dargemont C (2004). Nuclear import of HIV-1 integrase is inhibited in vitro by styrylquinoline derivatives. Mol Pharmacol.

[B84] Hazuda DJ, Young SD, Guare JP, Anthony NJ, Gomez RP, Wai JS, Vacca JP, Handt L, Motzel SL, Klein HJ (2004). Integrase inhibitors and cellular immunity suppress retroviral replication in rhesus macaques. Science.

[B85] Hazuda DJ, Anthony NJ, Gomez RP, Jolly SM, Wai JS, Zhuang L, Fisher TE, Embrey M, Guare JP, Egbertson MS (2004). A naphthyridine carboxamide provides evidence for discordant resistance between mechanistically identical inhibitors of HIV-1 integrase. Proc Natl Acad Sci USA.

[B86] Fikkert V, Van Maele B, Vercammen J, Hantson A, Van Remoortel B, Michiels M, Gurnari C, Pannecouque C, De Maeyer M, Engelborghs Y (2003). Development of resistance against diketo derivatives of human immunodeficiency virus type 1 by progressive accumulation of integrase mutations. J Virol.

[B87] Gerton JL, Brown PO (1997). The core domain of HIV-1 integrase recognizes key features of its DNA substrates. J Biol Chem.

[B88] Laboulais C, Deprez E, Leh H, Mouscadet JF, Brochon JC, Le Bret M (2001). HIV-1 integrase catalytic core: molecular dynamics and simulated fluorescence decays. Biophys J.

[B89] Vink C, van Gent DC, Elgersma Y, Plasterk RH (1991). Human immunodeficiency virus integrase protein requires a subterminal position of its viral DNA recognition sequence for efficient cleavage. J Virol.

[B90] Lee I, Harshey RM (2001). Importance of the conserved CA dinucleotide at Mu termini. J Mol Biol.

[B91] Delelis O, Petit C, Leh H, Mbemba G, Mouscadet JF, Sonigo P (2005). A novel function for spumaretrovirus integrase: an early requirement for integrase-mediated cleavage of 2 LTR circles. Retrovirology.

[B92] Delelis O, Lehmann-Che J, Saib A (2004). Foamy viruses – a world apart. Curr Opin Microbiol.

[B93] Chun TW, Carruth L, Finzi D, Shen X, DiGiuseppe JA, Taylor H, Hermankova M, Chadwick K, Margolick J, Quinn TC (1997). Quantification of latent tissue reservoirs and total body viral load in HIV-1 infection. Nature.

[B94] Svarovskaia ES, Barr R, Zhang X, Pais GC, Marchand C, Pommier Y, Burke TR, Pathak VK (2004). Azido-containing diketo acid derivatives inhibit human immunodeficiency virus type 1 integrase in vivo and influence the frequency of deletions at two-long-terminal-repeat-circle junctions. J Virol.

[B95] Parissi V, Calmels C, de Soultrait VR, Caumont A, Fournier M, Chaignepain S, Litvak S (2001). Functional interactions of human immunodeficiency virus type 1 integrase with human and yeast HSP60. J Virol.

[B96] Busschots K, Vercammen J, Emiliani S, Benarous R, Engelborghs Y, Christ F, Debyser Z (2005). The interaction of LEDGF/p75 with integrase is lentivirus-specific and promotes DNA binding. J Biol Chem.

[B97] McKee CJ, Kessl JJ, Shkriabai N, Dar MJ, Engelman A, Kvaratskhelia M (2008). Dynamic Modulation of HIV-1 Integrase Structure and Function by Cellular Lens Epithelium-derived Growth Factor (LEDGF) Protein. J Biol Chem.

[B98] Emiliani S, Mousnier A, Busschots K, Maroun M, Van Maele B, Tempe D, Vandekerckhove L, Moisant F, Ben Slama L, Witvrouw M (2005). Integrase mutants defective for interaction with LEDGF/p75 are impaired in chromosome tethering and HIV-1 replication. J Biol Chem.

[B99] Pruss D, Reeves R, Bushman FD, Wolffe AP (1994). The influence of DNA and nucleosome structure on integration events directed by HIV integrase. J Biol Chem.

[B100] Pruss D, Bushman FD, Wolffe AP (1994). Human immunodeficiency virus integrase directs integration to sites of severe DNA distortion within the nucleosome core. Proc Natl Acad Sci USA.

[B101] Taganov KD, Cuesta I, Daniel R, Cirillo LA, Katz RA, Zaret KS, Skalka AM (2004). Integrase-specific enhancement and suppression of retroviral DNA integration by compacted chromatin structure in vitro. J Virol.

[B102] Wang T, Balakrishnan M, Jonsson CB (1999). Major and minor groove contacts in retroviral integrase-LTR interactions. Biochemistry.

[B103] Lin CW, Engelman A (2003). The barrier-to-autointegration factor is a component of functional human immunodeficiency virus type 1 preintegration complexes. J Virol.

[B104] Chen H, Engelman A (1998). The barrier-to-autointegration protein is a host factor for HIV type 1 integration. Proc Natl Acad Sci USA.

[B105] Harris D, Engelman A (2000). Both the structure and DNA binding function of the barrier-to-autointegration factor contribute to reconstitution of HIV type 1 integration in vitro. J Biol Chem.

[B106] Li L, Yoder K, Hansen MS, Olvera J, Miller MD, Bushman FD (2000). Retroviral cDNA integration: stimulation by HMG I family proteins. J Virol.

[B107] Kalpana GV, Marmon S, Wang W, Crabtree GR, Goff SP (1994). Binding and stimulation of HIV-1 integrase by a human homolog of yeast transcription factor SNF5. Science.

[B108] Maroun M, Delelis O, Coadou G, Bader T, Segeral E, Mbemba G, Petit C, Sonigo P, Rain JC, Mouscadet JF (2006). Inhibition of early steps of HIV-1 replication by SNF5/Ini1. J Biol Chem.

[B109] Boese A, Sommer P, Gaussin A, Reimann A, Nehrbass U (2004). Ini1/hSNF5 is dispensable for retrovirus-induced cytoplasmic accumulation of PML and does not interfere with integration. FEBS Lett.

[B110] Ariumi Y, Serhan F, Turelli P, Telenti A, Trono D (2006). The integrase interactor 1 (INI1) proteins facilitate Tat-mediated human immunodeficiency virus type 1 transcription. Retrovirology.

[B111] Yung E, Sorin M, Wang EJ, Perumal S, Ott D, Kalpana GV (2004). Specificity of interaction of INI1/hSNF5 with retroviral integrases and its functional significance. J Virol.

[B112] Zheng R, Ghirlando R, Lee MS, Mizuuchi K, Krause M, Craigie R (2000). Barrier-to-autointegration factor (BAF) bridges DNA in a discrete, higher-order nucleoprotein complex. Proc Natl Acad Sci USA.

[B113] Lewinski MK, Bushman FD (2005). Retroviral DNA integration – mechanism and consequences. Adv Genet.

[B114] Llano M, Vanegas M, Fregoso O, Saenz D, Chung S, Peretz M, Poeschla EM (2004). LEDGF/p75 determines cellular trafficking of diverse lentiviral but not murine oncoretroviral integrase proteins and is a component of functional lentiviral preintegration complexes. J Virol.

[B115] Cherepanov P, Ambrosio AL, Rahman S, Ellenberger T, Engelman A (2005). Structural basis for the recognition between HIV-1 integrase and transcriptional coactivator p75. Proc Natl Acad Sci USA.

[B116] Shun MC, Raghavendra NK, Vandegraaff N, Daigle JE, Hughes S, Kellam P, Cherepanov P, Engelman A (2007). LEDGF/p75 functions downstream from preintegration complex formation to effect gene-specific HIV-1 integration. Genes Dev.

[B117] Lee MS, Craigie R (1998). A previously unidentified host protein protects retroviral DNA from autointegration. Proc Natl Acad Sci USA.

[B118] Masson C, Bury-Mone S, Guiot E, Saez-Cirion A, Schoevaert-Brossault D, Brachet-Ducos C, Delelis O, Subra F, Jeanson-Leh L, Mouscadet JF (2007). Ku80 participates in the targeting of retroviral transgenes to the chromatin of CHO cells. J Virol.

[B119] Jacque JM, Stevenson M (2006). The inner-nuclear-envelope protein emerin regulates HIV-1 infectivity. Nature.

[B120] Cereseto A, Manganaro L, Gutierrez MI, Terreni M, Fittipaldi A, Lusic M, Marcello A, Giacca M (2005). Acetylation of HIV-1 integrase by p300 regulates viral integration. EMBO J.

[B121] Topper M, Luo Y, Zhadina M, Mohammed K, Smith L, Muesing MA (2007). Posttranslational acetylation of the human immunodeficiency virus type 1 integrase carboxyl-terminal domain is dispensable for viral replication. J Virol.

[B122] Leavitt AD, Robles G, Alesandro N, Varmus HE (1996). Human immunodeficiency virus type 1 integrase mutants retain in vitro integrase activity yet fail to integrate viral DNA efficiently during infection. J Virol.

[B123] Tsurutani N, Kubo M, Maeda Y, Ohashi T, Yamamoto N, Kannagi M, Masuda T (2000). Identification of critical amino acid residues in human immunodeficiency virus type 1 IN required for efficient proviral DNA formation at steps prior to integration in dividing and nondividing cells. J Virol.

[B124] Lu R, Limon A, Devroe E, Silver PA, Cherepanov P, Engelman A (2004). Class II integrase mutants with changes in putative nuclear localization signals are primarily blocked at a postnuclear entry step of human immunodeficiency virus type 1 replication. J Virol.

[B125] Depienne C, Mousnier A, Leh H, Le Rouzic E, Dormont D, Benichou S, Dargemont C (2001). Characterization of the nuclear import pathway for HIV-1 integrase. J Biol Chem.

[B126] Ao Z, Huang G, Yao H, Xu Z, Labine M, Cochrane AW, Yao X (2007). Interaction of human immunodeficiency virus type 1 integrase with cellular nuclear import receptor importin 7 and its impact on viral replication. J Biol Chem.

[B127] de Soultrait VR, Caumont A, Durrens P, Calmels C, Parissi V, Recordon P, Bon E, Desjobert C, Tarrago-Litvak L, Fournier M (2002). HIV-1 integrase interacts with yeast microtubule-associated proteins. Biochim Biophys Acta.

[B128] Arhel N, Genovesio A, Kim KA, Miko S, Perret E, Olivo-Marin JC, Shorte S, Charneau P (2006). Quantitative four-dimensional tracking of cytoplasmic and nuclear HIV-1 complexes. Nat Methods.

[B129] Bukrinsky M (2004). A hard way to the nucleus. Mol Med.

[B130] Bouyac-Bertoia M, Dvorin JD, Fouchier RA, Jenkins Y, Meyer BE, Wu LI, Emerman M, Malim MH (2001). HIV-1 infection requires a functional integrase NLS. Mol Cell.

[B131] Dvorin JD, Bell P, Maul GG, Yamashita M, Emerman M, Malim MH (2002). Reassessment of the roles of integrase and the central DNA flap in human immunodeficiency virus type 1 nuclear import. J Virol.

[B132] Christ F, Thys W, De Rijck J, Gijsbers R, Albanese A, Arosio D, Emiliani S, Rain JC, Benarous R, Cereseto A (2008). Transportin-SR2 imports HIV into the nucleus. Curr Biol.

[B133] Mousnier A, Kubat N, Massias-Simon A, Segeral E, Rain JC, Benarous R, Emiliani S, Dargemont C (2007). von Hippel Lindau binding protein 1-mediated degradation of integrase affects HIV-1 gene expression at a postintegration step. Proc Natl Acad Sci USA.

[B134] Studamire B, Goff SP (2008). Host proteins interacting with the Moloney murine leukemia virus integrase: multiple transcriptional regulators and chromatin binding factors. Retrovirology.

